# RBM10 inhibits pancreatic cancer development by suppressing immune escape through PD-1 expression

**DOI:** 10.7150/jca.111459

**Published:** 2025-07-04

**Authors:** Xia Gao, Xiuqin Zhang, Junjie Huang, Zhenyu Tan, Jing Yang, Liuhong Yuan, Pengjun Wang, Feier Chen, Huiyan Wu, Changyi Feng, Hong Yu, Shisan Bao, Da Fu, Kun Tao

**Affiliations:** 1 Department of Pathology, Tongren Hospital, Shanghai Jiao Tong University School of Medicine, Shanghai, China.; 2 Department of Pathology, Tongji Hospital, Tongji University School of Medicine, Shanghai, China.; 3 Research Institute of Pancreatic Diseases, Shanghai Key Laboratory of Translational Research for Pancreatic Neoplasms, Shanghai Jiaotong University School of Medicine, Shanghai 200025, China.; 4 Department of Pathology, The Affiliated Taizhou Peoples Hospital of Nanjing Medical University, Taizhou 225300, Jiangsu, China.; 5 Department of Pathology, Taizhou School of Clinical Medicine, Nanjing Medical University, Taizhou 225300, Jiangsu, China.

**Keywords:** PAAD, RBM10, JAK-STAT, PD-1, NK cell.

## Abstract

RNA-binding motif protein-10 (RBM10) plays a role in pancreatic adenocarcinoma (PAAD), though its precise underlying mechanism remains unclear. The current study investigates the role of RBM10 in pancreatic cancer progression and immune regulation.

RBM10 expression in pancreatic tissues from PAAD patient was assessed using Western blotting, RT-qPCR, and immunohistochemistry, revealing lower levels in pancreatic cancerous tissues compared to adjacent non-cancerous tissues. This finding aligns with *in vitro* experiments where RBM10 knockdown in pancreatic cancer cells enhanced colony formation, migration, and proliferation, which correlated with increased P-JAK1, P‑JAK2, and P-STAT3 levels. Bioinformatics identified RBM10-related pathways and immune changes. Moreover, RBM10 deficiency in cancer cells increased PD-1 expression in natural killer cells *in vitro*, reducing their tumour-killing ability. However, treatment with the JAK pathway inhibitor AZD1480 restored NK cell cytotoxicity against cancer cells.

Finally, high RBM10 expression was associated with a favourable prognosis in pancreatic cancer patients, suggesting that RBM10 inhibits pancreatic cancer progression by suppressing tumour immune escape through JAK-STAT-mediated regulation of PD-1 expression in NK cells. This finding offers potential for the development of novel precision-targeted therapies in the management of pancreatic cancer.

## Introduction

Pancreatic cancer is a highly fatal disease with a poor 5-year survival rate, making it the second leading cause of cancer-related deaths, despite its relatively low incidence 1. It often presents with ambiguous symptoms, making early diagnosis challenging. This is partly due to the lack of obvious symptoms and the absence of reliable diagnostic markers with high sensitivity and specificity 2.

Current management of pancreatic cancer includes surgery, radiotherapy and immunotherapy 3. Surgery remains the only potential cure, but only 20% of patients are eligible for surgical intervention with reasonably good outcomes, though the risk of recurrence remains extremely high 2, 4. More recently, advances in molecular biology have provided insights into the occurrence and development of pancreatic cancer, with a particular focus on key biomarkers for targeted therapy, which have garnered extensive attention 5, 6.

RNA-binding proteins (RBPs) are crucial intracellular regulators involved in RNA splicing, translocation, localisation, and translation 7. Among them, the RNA-binding motif (RBM) protein family participates in diverse RNA metabolic processes, including splicing, translation, and stability, with aberrant expression linked to cancer development 7. Specific RBM proteins exhibit distinct roles in cancer. For instance, RBM5 overexpression induces autophagy and suppresses lung adenocarcinoma by modulating Beclin1, NF-kB/P65, and Bcl-2 8. RBM38 inhibits the progression of colorectal cancer by interacting with the PTEN 3'UTR and miR-92a-3p 9. RBM43 negatively regulates cyclin B1 mRNA, inhibiting hepatocellular carcinoma progression 10. Conversely, RBM4 promotes angiogenesis in hepatocellular carcinoma *via* NF-kB activation, as well as upregulation of VEGF-A 11, while RBM7 stabilises CDK1 mRNA, enhancing breast cancer proliferation 12. These findings suggest that RBM proteins play differential roles, i.e. act as either tumour suppressors or promoters, depending on the context.

RBM10 is a critical member of the RBM protein family. It primarily regulates gene transcription through alternative splicing and impacts cellular processes such as proliferation and apoptosis 13. Mutations in the RBM10 gene are closely linked to TARP syndrome, highlighting its pivotal role in various cancers. RBM10 inhibits cellular proliferation *via* the RAP1/AKT/CREB signalling pathway in lung adenocarcinoma 14. RBM10 deletion is associated with resistance to EGFR inhibitors in EGFR-mutant lung cancer, likely due to a reduced BclxS/BclxL ratio, which diminishes apoptosis induced by EGFR inhibitors 15. There is a correlation between RBM10 expression and survival in lung adenocarcinoma patients. RBM10 mutations frequently coexist with EGFR mutations, influencing lung adenocarcinoma progression through gene mutation and splicing regulation. In RBM10-deficient lung adenocarcinoma, combining a spliceosome inhibitor with the EGFR tyrosine kinase inhibitor (Osimertinib) improves therapeutic efficacy and reduces drug resistance, offering a promising treatment strategy 16, 17.

Furthermore, RBM10 is suggested to be a tumour suppressor, with single-gene somatic mutations linked to shorter progression-free survival in metastatic colorectal cancer patients 18. Downregulated RBM10 in malignant tissues, particularly in advanced-stage tumours, is linked with poorer prognosis for hepatocellular carcinoma patients 19. Similarly, low RBM10 expression is associated with poorer survival in breast cancer. RBM10 deletion significantly enhances breast cancer proliferation and migration, leading to accelerated tumour growth in nude mice *in vivo* 20. RBM10 induces apoptosis in U2OS cells (osteosarcoma) by suppressing Bcl2 expression, promoting caspase-3 activity, and increasing TNF production 21. Overexpression of RBM10 reduces proliferation, colony formation, migration, and invasion of osteosarcoma cells 21.

Although RBM10 has been shown to play an anti-tumour role in pancreatic cancer 22, 23, its precise underlying mechanisms remain poorly understood. Therefore, we aimed to investigate the biological function of RBM10 in pancreatic cancer and the potential mechanisms through which it operates.

## Materials and Methods

### Data processing and analysis

We downloaded mRNA transcriptome data and clinical information for 178 pancreatic adenocarcinoma (PAAD) patients from TCGA and data for 135 PAAD patients from CPTAC. We compiled a gene set for the RBM family, consisting of 112 genes. Tumour samples were categorised into RBM10 high and low expression groups, based on the optimal cutoff from RBM10 survival analysis. Differentially expressed mRNAs (DEmRNAs) were identified with P < 0.05 and |FC| > 2. Clinical data from TCGA were also analysed to explore the correlation between RBM10 expression and clinical indicators.

### Sample collection

We collected samples of cancerous and paraneoplastic tissues from 30 PAAD patients at Ruijin Hospital, Shanghai Jiao Tong University School of Medicine. The inclusion criteria: 1. Age 18-75 years with pathologically confirmed diagnosis of PAAD; 2. No combination of other malignant tumors; The exclusive criteria: 1. Suffering from autoimmune disease; 2. Undergoing immunosuppressive therapy. Informed consent was obtained from each patient prior to surgery, with patients being informed that, in addition to the diagnostic purpose, some of the samples would also be used for medical research in a de-identified manner. The study protocol was approved by the Ethics Committee of Ruijin Hospital, Shanghai Jiao Tong University School of Medicine, as described 24.

### RNA extraction and Real-time quantitative reverse transcription PCR (RTq-PCR)

Total RNA from tissues or cells was extracted using the SteadyPure Rapid RNA Extraction Kit (Accurate Biology, China). RNA purity and concentration were measured, and the RNA was subsequently reverse transcribed into cDNA using the HiScript III All-in-one RT SuperMix Perfect for qPCR (Vazyme, China). The qPCR assay was performed using the ChamQ SYBR qPCR Master Mix (Vazyme, China). The primer sequences are provided in Table S1.

### Western blotting

Western blot was performed as described previously 25. Briefly, total protein was extracted by lysing cells with RIPA buffer (Epizyme Biomedical, China) containing protease and phosphatase inhibitors. Proteins were separated by SDS-PAGE, transferred to PVDF membranes, and incubated overnight with the primary antibody at 4°C. After washing with TBST, the secondary antibody was applied and incubated for 1 hour at room temperature. Protein bands were detected using the Tanon-5200 chemiluminescence system (Tanon, China), and gray-scale analysis was performed with ImageJ software. Primary antibody details are in Table S2.

### Cell culture

PANC-1 and PATU-8988 (human pancreatic cancer cell lines), and HEK-293T (a human embryonic kidney cell line) were purchased from the Cell Resource Centre of Shanghai Institutes for Biotechnology, Chinese Academy of Sciences. NK96MI (NK cell line) cells were obtained from HyCyte (Suzhou, China). PANC-1, PATU-8988, and HEK-293T cells were cultured in DMEM medium supplemented with 10% foetal bovine serum and 1% penicillin/streptomycin 26. NK96MI cells were cultured in specialized complete medium (HyCyte, China) 27. All cells were maintained at 37 ^o^C in a humidified incubator with 5% CO₂.

### Cell Transfection and Stable Cell Line Generation

HEK-293T cells were seeded in 6-well plates, and when cell confluence reached ~70%, the medium was replaced with fresh 2 mL of DMEM complete medium per well. The transfection mixture (125 μL DMEM + 2.5 μg DNA at PMD2G:psPAX2:target plasmid ratio of 3:2:4 + 4 μL Lipo8000™ Transfection Reagent, Beyotime, China) was prepared, gently mixed, and added to each well. The medium was refreshed every 8 hours. PATU-8988 and PANC-1 cells seeded at ~50% confluence were transduced with 1 mL of supernatant, 1 mL of DMEM complete medium, and 2 μL polybrene (Beyotime, China) per well. After observing transfection efficiency under a microscope, the medium was replaced, and puromycin was added for selection, as described 28. The plasmid sequences are provided in Table S3.

### CCK-8 assay

The transfected cells were resuspended in 100 μL of DMEM complete medium at a density of 1 x 10³ cells/well and seeded into 96-well plates. Cell viability was assessed every 24 hours using the Cell Counting Kit-8 (CCK-8, Meilunbio, China). After incubating the cells with the CCK-8 reagent for 2 hours at 37 °C, the absorbance at 450 nm was measured using a microplate reader. Measurements were recorded daily for 5 consecutive days, as described 29.

### Colony formation assay

The transfected cells were seeded at 1×10³ cells/well in 6-well plates and cultured for 7-14 days, with medium changes and cell monitoring every 3 days. Upon colony formation, the cells were washed with PBS, fixed with 4% paraformaldehyde for 30 minutes, stained with 1 mL of crystal violet for 10 minutes, rinsed with water, air-dried, and photographed for colony counting.

### Transwell migration assay

Transfected cells were digested, centrifuged, and resuspended in serum-free DMEM at a concentration of 1×10⁵ cells/mL. A 24-well plate was prepared with 500 μL of DMEM complete medium per well, followed by insertion of Corning chambers (300 μL of cell suspension per chamber). After 24 hours of incubation, cells were fixed with 4% paraformaldehyde for 30 minutes, washed with PBS, and stained with crystal violet for 10 minutes. Residual stain on the inner chamber surfaces was removed with a cotton swab, and the chambers were air-dried. Cells were then observed under a microscope and photographed for counting, as described 29.

### Cytotoxicity assay

Tumour cells were co-cultured with NK92MI cells, and their cytotoxicity was assessed using the Cytotoxicity LDH Assay Kit (MCE, USA), as described 30. Each group included six replicate wells, and the results were analysed and plotted using GraphPad Prism (version 8.3.0).

### Functional enrichment analysis of DemRNAs

PAAD patients from the TCGA and CPTAC databases were grouped by high and low RBM10 expression. Differential gene analysis was conducted using the limma package in R (4.3.1) with thresholds of |FC|>2 and P<0.05. The resulting gene lists were uploaded to the DAVID database for Gene Ontology (GO) and KEGG pathway analyses, visualized with the R package "ggplot2". Gene set enrichment analysis (GSEA) of RBM10 was also performed and visualized using R.

### Animals

RBM10^fl/fl^/Pdx1-cre mice and C57BL/6J mice were purchased from the Model Organisms Centre (Shanghai, China). The mice were housed in cages (6 mice per cage) under SPF conditions, with a temperature of 24°C, relative humidity of 35% *ad libitum*. All experimental procedures were approved by the Ethics Committee, Tongren Hospital, Shanghai Jiao Tong University School of Medicine.

### Immune-related analysis

To investigate the relationship between RBM10 expression and tumour immunity, we analysed the infiltration of 22 immune cells using CIBERSORTx (https://cibersortx.stanford.edu/) [31]and visualized the results with R software. Immune scores, including “StromalScore,” “ImmuneScore,” and “ESTIMATEScore,” were obtained from Sangerbox (http://sangerbox.com/) to assess their correlation with RBM10^32^. The relationships between RBM10 and immunoregulatory and immune checkpoint genes were explored using respective analysis modules. Additionally, we used TIMER (https://cistrome.shinyapps.io/timer/) to analyse the association between RBM10 gene copy number and immune cell infiltration 33.

### Immunohistochemistry (IHC)

The collected tissues were fixed in 4% paraformaldehyde for 24 hours, embedded in paraffin, and cut into 4 μm thick sections. IHC was performed as described previously 34-36. The immunolabeled slides were observed under a microscope, and images were captured.

### Multiplex immunohistochemistry (mIHC)

Multiplex immunohistochemistry was performed as described 37. Briefly deparaffined sections were treated and incubated with primary antibody overnight at 4°C. The next day, addition of secondary antibody was incubated for 30 min at room temperature followed by addition of fluorescein for 20 min. After antigen repair again the blocking solution was incubated for 2 hours at room temperature, the primary antibody was replaced and incubated at 4°C overnight. On the third day, dropwise addition of secondary antibody was incubated at room temperature for 30 minutes and then fluorescein was replaced and incubated at room temperature for 20 minutes, and dropwise addition of DAPI dye-containing sealer (Beyotime, China) was performed for sealing treatment. The staining results were observed under fluorescence microscope and images were recorded. A multiple fluorescent staining kit (AiFang biological, China) was used.

### Statistical analysis

Statistics were performed as described 34-36, 38. Differential expression was analyzed using R software (4.3.1) and GraphPad Prism (8.3.0). Unpaired t-test, one-way ANOVA, and chi-square test were applied to assess group differences, with P < 0.05 considered statistically significant.

## Results

### RBM10 is lowly expressed in PAAD and associated with favourable patient prognosis

**Figure 1:**
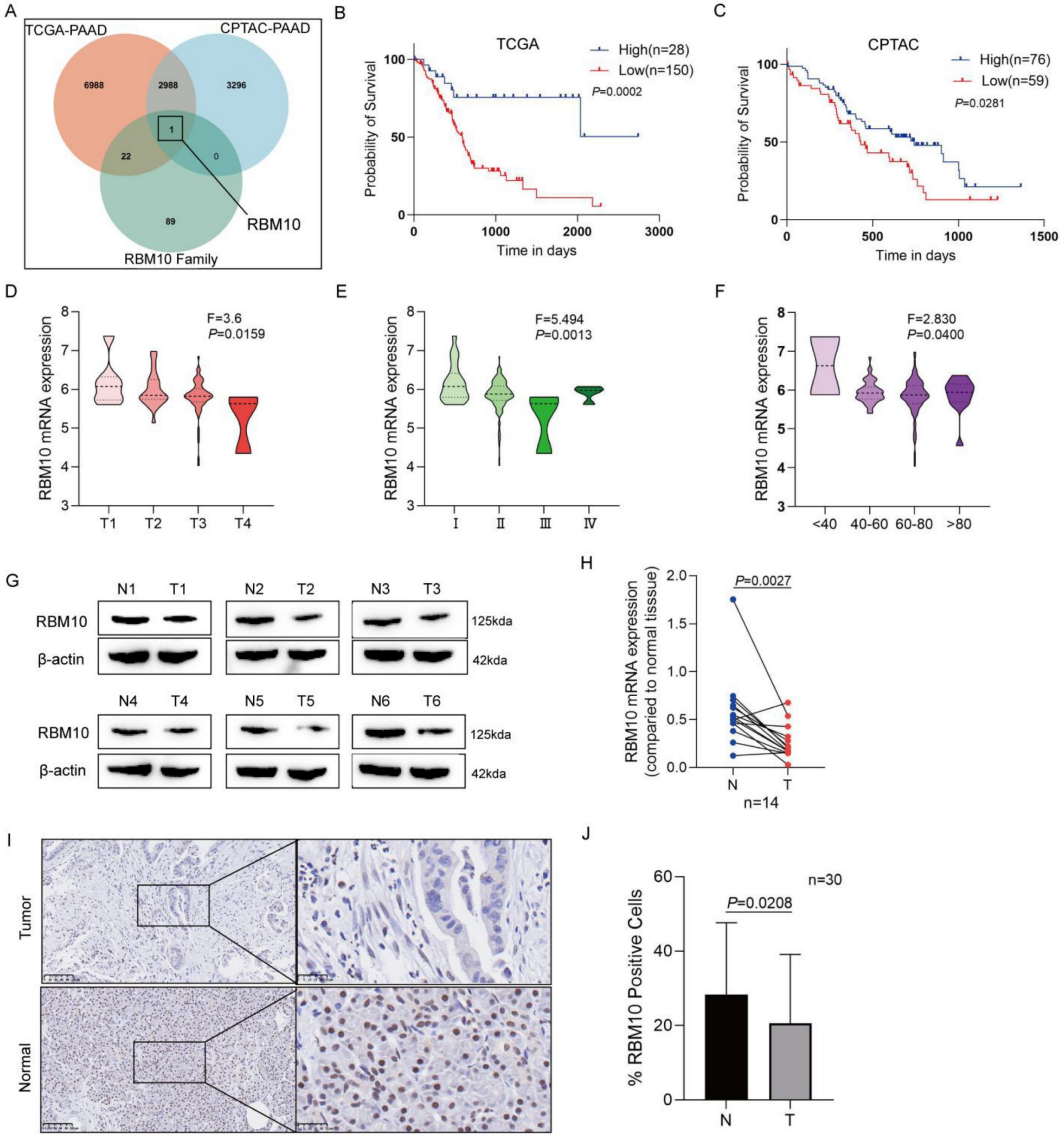
** RBM10 is lowly expressed in PAAD and associated with favourable patient prognosis. A.** Venn diagram showing the intersection of PAAD-related survival analysis results with RBM protein family genes in the TCGA and CPTAC databases; **B.** Kaplan-Meier curve of overall survival based on RBM10 expression in PAAD patients in the TCGA database; **C**. Kaplan-Meier curve of overall survival based on RBM10 expression in PAAD patients in the CPTAC database; **D-F.** Clinicopathological features of PAAD associated with RBM10 expression in the TCGA database: T stage (D), clinical stage (E), age (F); **G.** Western blot detection of RBM10 expression in cancerous and para-cancerous normal tissues from 6 PAAD patients; **H.** RT-qPCR detection of RBM10 expression in cancerous and para-cancerous tissues from 14 PAAD patients; **I-J.** Representative images and quantitative results of IHC staining of RBM10 in cancerous and para-cancerous tissues from PAAD patients. Scale bar: 20 μm.

Using the dataset of 178 PAAD patients from the TCGA database, we performed survival analysis with the optimal cutoff value calculated in R software (CUTOFF > 0, P < 0.05), identifying 12,126 candidate genes. The same method was applied to the dataset of 135 PAAD patients from the CPTAC database, resulting in the identification of 6,285 candidate genes. These two datasets were intersected with the RBM family gene set (containing 112 genes), leading to the final selection of our target, RBM10 (Figure 1A). The optimal cutoff value for RBM10 expression was calculated in R software, and tumour samples were divided into RBM10 high and low expression groups. Survival analysis revealed that patients with low RBM10 expression had worse survival (P < 0.05) (Figure 1B, C).

Using data from PAAD patients in the TCGA database, RBM10 expression was found to be associated with patients' T stage, clinical stage, and age (all P < 0.05) (Figure 1D-F), but not with lymph node metastasis, distant metastasis, or sex (Figure S1).

Western blot analysis of cancerous and para-cancerous tissues from PAAD patients revealed reduced RBM10 expression in cancerous tissues (Figure 1G). The protein expression levels of RBM10 were consistent with RT-qPCR analysis of 14 pairs of patient cancer and para-cancerous tissues, showing that RBM10 mRNA levels in PAAD tissues were lower than in para-cancerous tissues (P < 0.01) (Figure 1H).

Additionally, IHC staining of 30 pairs of cancerous and para-cancerous tissues from PAAD patients showed that RBM10 expression was reduced by approximately 15% in pancreatic cancer tissues compared to para-cancerous tissues (P < 0.05) (Figure 1I, J).

### RBM10 deficiency promotes proliferation and migration of pancreatic cancer cells

**Figure 2:**
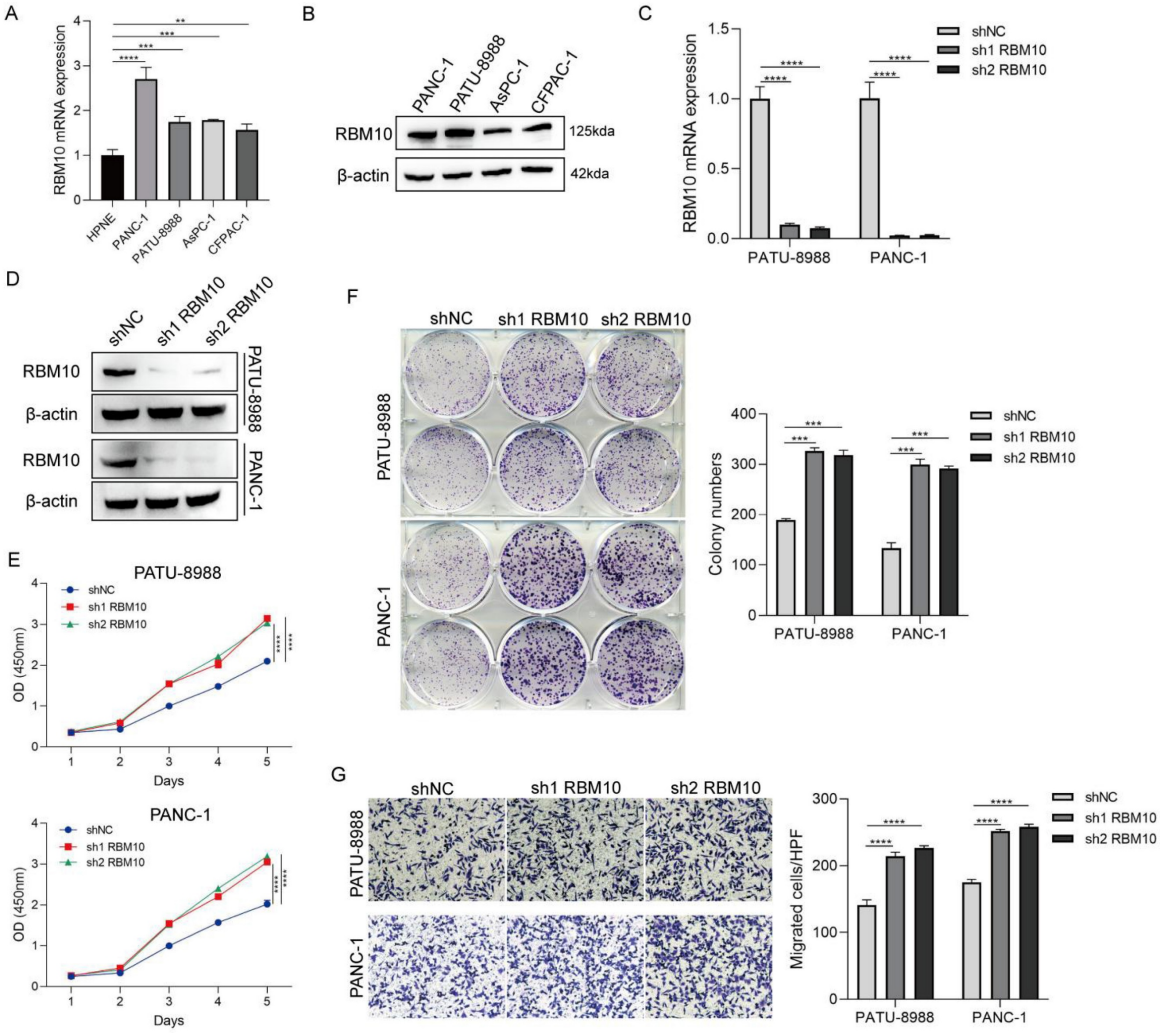
** Deletion of RBM10 promotes proliferation and migration of pancreatic cancer cells. A-B.** Expression levels of RBM10 in common pancreatic cancer cell lines, as determined by RT-qPCR (A) and Western blot (B); **C-D.** Validation of RBM10 knockdown in PATU-8988 and PANC-1 cells, as assessed by RT-qPCR (C) and Western blot (D); **E.** CCK-8 assay showing cell viability after RBM10 knockdown in PATU-8988 and PANC-1 cells; **F.** Colony formation assay after RBM10 knockdown in PATU-8988 and PANC-1 cells; **G.** Transwell migration assay after RBM10 knockdown in PATU-8988 and PANC-1 cells.

To explore the role of RBM10 in pancreatic cancer, RBM10 knockdown was performed in pancreatic cancer cell lines. RT-qPCR and Western blot analyses of these manipulated cells showed that the expression of RBM10 was higher in PATU-8988 and PANC-1 pancreatic cancer cells compared to other cancer cell lines (Fig. 2A, B). Therefore, PATU-8988 and PANC-1 cells were selected for subsequent experiments. Successful transfection of the cells was confirmed by RT-qPCR and Western blot, showing effective RBM10 knockdown (Fig. 2C, D). The CCK-8 assay showed a significant increase, approximately 1.5 times, in the viability of PATU-8988 and PANC-1 cells following RBM10 knockdown, suggesting successful knockdown and promotion of pancreatic cancer cell proliferation (P < 0.05) (Fig. 2E). Colony formation assays revealed a marked increase, approximately 1.8 times, in the number of colonies formed by PATU-8988 and PANC-1 cells after RBM10 knockdown (P < 0.05) (Fig. 2F). Transwell migration assays showed that the migration of PATU-8988 and PANC-1 cells was significantly enhanced, nearly 1.5-fold, after RBM10 knockdown compared to the control group (P < 0.05) (Fig. 2G).

### Analysis of RBM10-related functional enrichment in PAAD

**Figure 3:**
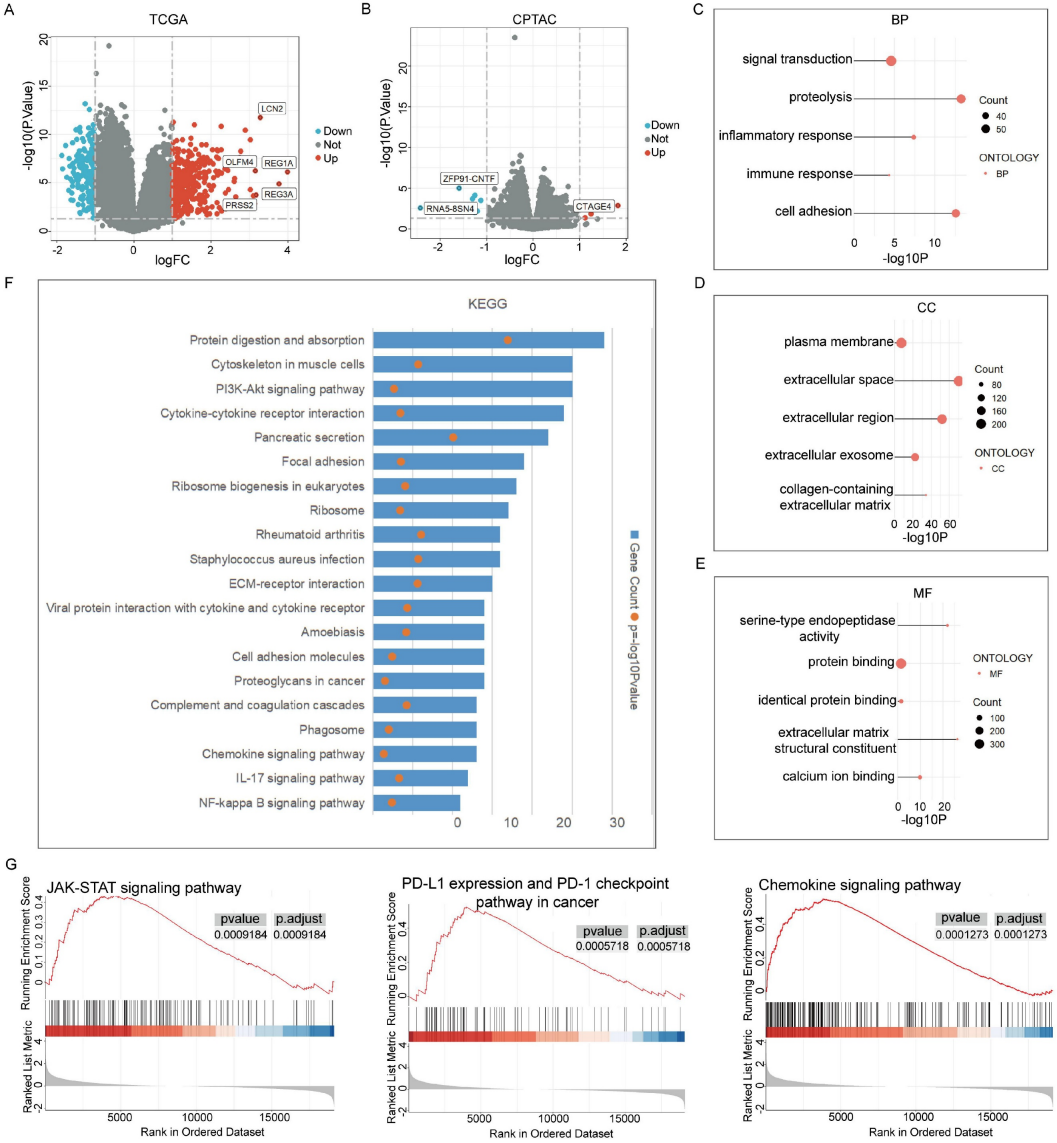
** Enrichment analysis of RBM10-related functions in pancreatic adenocarcinoma. A.** Volcano plot of differential gene expression between high and low RBM10 expression groups in pancreatic adenocarcinoma patients from the TCGA database. **B.** Volcano plot of differential gene expression between high and low RBM10 expression groups in pancreatic adenocarcinoma patients from the CPTAC database. **C-E.** Gene Ontology (GO) analysis of differential genes: Biological Processes (C), Cellular Components (D), and Molecular Functions (E). **F.** KEGG pathway analysis of differential genes. **G.** Gene Set Enrichment Analysis of high and low RBM10 expression groups.

To understand the role of RBM10 in pancreatic adenocarcinoma, functional enrichment analysis of RBM10-related genes was performed using pancreatic adenocarcinoma samples from the TCGA and CPTAC databases. In the TCGA database, 28 cases exhibited high RBM10 expression, while 150 cases exhibited low RBM10 expression, leading to the identification of 568 differential genes (|FC| > 1, P < 0.05), including 406 significantly upregulated and 162 significantly downregulated genes (Figure 3A). In the CPTAC database, 76 cases showed high RBM10 expression, and 59 cases showed low RBM10 expression, yielding 28 differential genes (|FC| > 1, P < 0.05), of which 5 were significantly upregulated and 23 were significantly downregulated (Figure 3B).

By combining the two sets of differential genes, we analysed the associated GO and KEGG pathways using the DAVID website (https://david.ncifcrf.gov/). These differential genes were primarily associated with biological processes related to cell-cell interactions and immune responses (Figure 3C), with notable alterations in cellular components such as extracellular regions and the plasma membrane (Figure 3D), and molecular functions related to protein binding (Figure 3E). KEGG pathway analysis revealed enrichment in multiple inflammatory and immune-related pathways (Figure 3F).

Additionally, GSEA analysis using R software identified enrichment in multiple cancer- and immune-related signalling pathways, including the JAK-STAT signalling pathway, PD-L1 expression and PD-1 checkpoint pathways, and the chemokine signalling pathway (Figure 3G).

### RBM10 deficiency affects the pancreas

**Figure 4:**
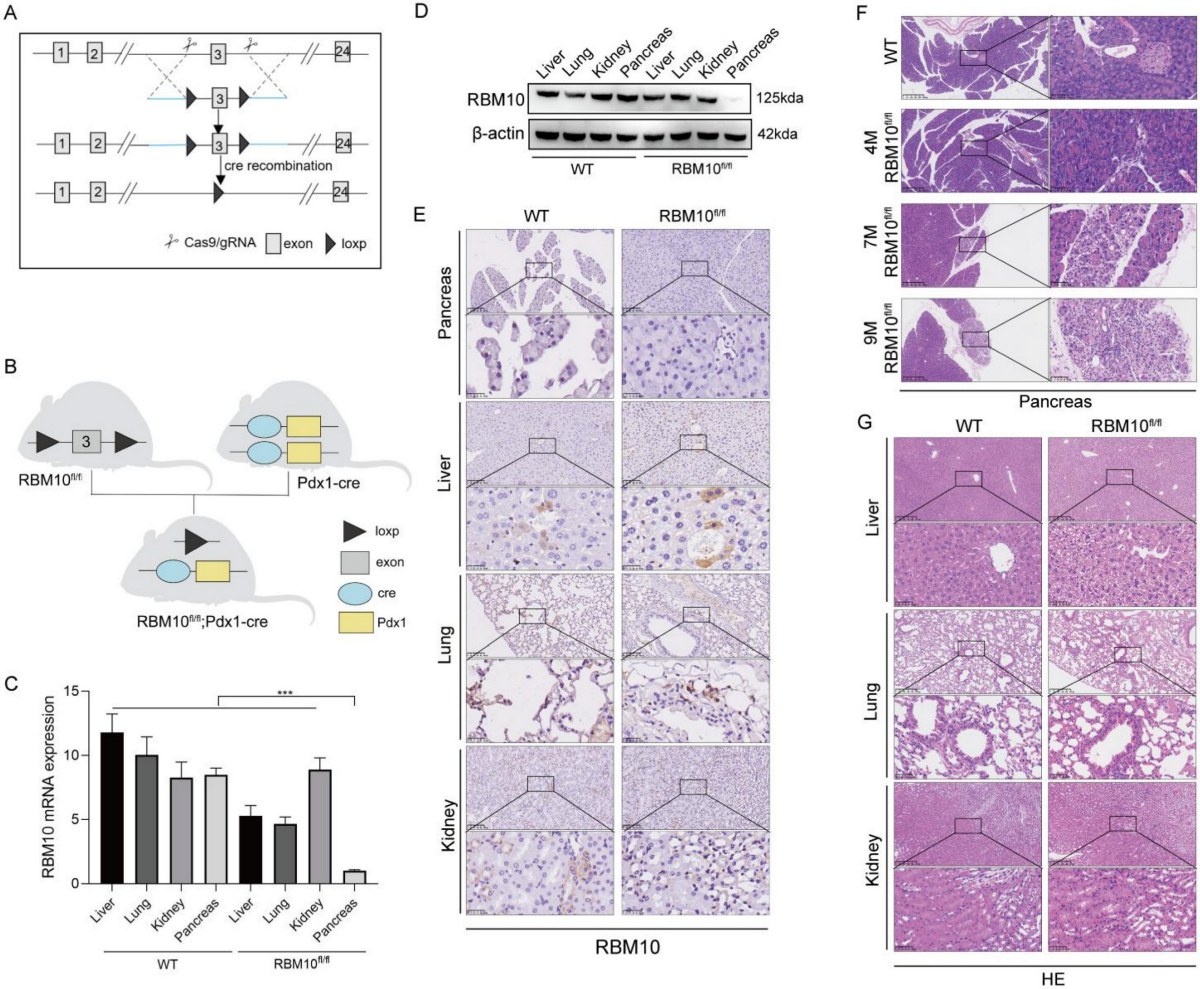
** RBM10 deletion affects the pancreas. A.** Schematic diagram of the RBM10 conditional knockout model; **B.** Schematic diagram of the RBM10^fl/fl^;Pdx1-cre mouse construct; **C.** RBM10 mRNA expression in various organs of RBM10^fl/fl^;Pdx1-cre mice and WT mice; **D.** RBM10 protein expression in various organs of RBM10^fl/fl^;Pdx1-cre mice and WT mice; **E.** RBM10 expression in various organs of RBM10^fl/fl^;Pdx1-cre mice and WT mice; **F.** Morphological structure of pancreatic tissue in RBM10^fl/fl^;Pdx1-cre mice at different time points; **G.** Morphological structure of various organs in RBM10^fl/fl^;Pdx1-cre mice and WT mice; Scale bar: 20μm.

To explore the role of RBM10 *in vivo*, we generated a pancreas-specific RBM10 knockout mouse model. LoxP sites were inserted into the non-coding region flanking exon 3 of the RBM10 gene to create RBM10 conditional knockout mice, which were subsequently crossed with pancreas-specific Pdx1-Cre mice to produce pancreas-specific RBM10 knockout mice (RBM10^fl/fl^;Pdx1-Cre) (Figure 4A, B). RT-qPCR (Figure 4C) and Western blot (Figure 4D) confirmed the specific deletion of RBM10 in pancreatic tissues, which was further validated by IHC staining (Figure 4E).

Histopathological analysis of the pancreas in RBM10^fl/fl^ mice showed localized reductions in acinar cells and an increase in ductal epithelial cells, accompanied by mild fibrosis and inflammatory cell infiltration at 7 months of age. These manifestations progressed by 9 months, although the structure of the ductal glands and the nuclear-to-cytoplasm ratio remained normal, suggesting that RBM10 deletion negatively impacts pancreatic tissue (Figure 4F).

No significant pathological changes were observed in other major organs of RBM10^fl/fl^;Pdx1-Cre mice compared to wild-type mice (Figure 4G). This indicates that the absence of RBM10 primarily affects the pancreas, leading to the development of certain lesions.

### Immunological characterization of RBM10 in PAAD

**Figure 5:**
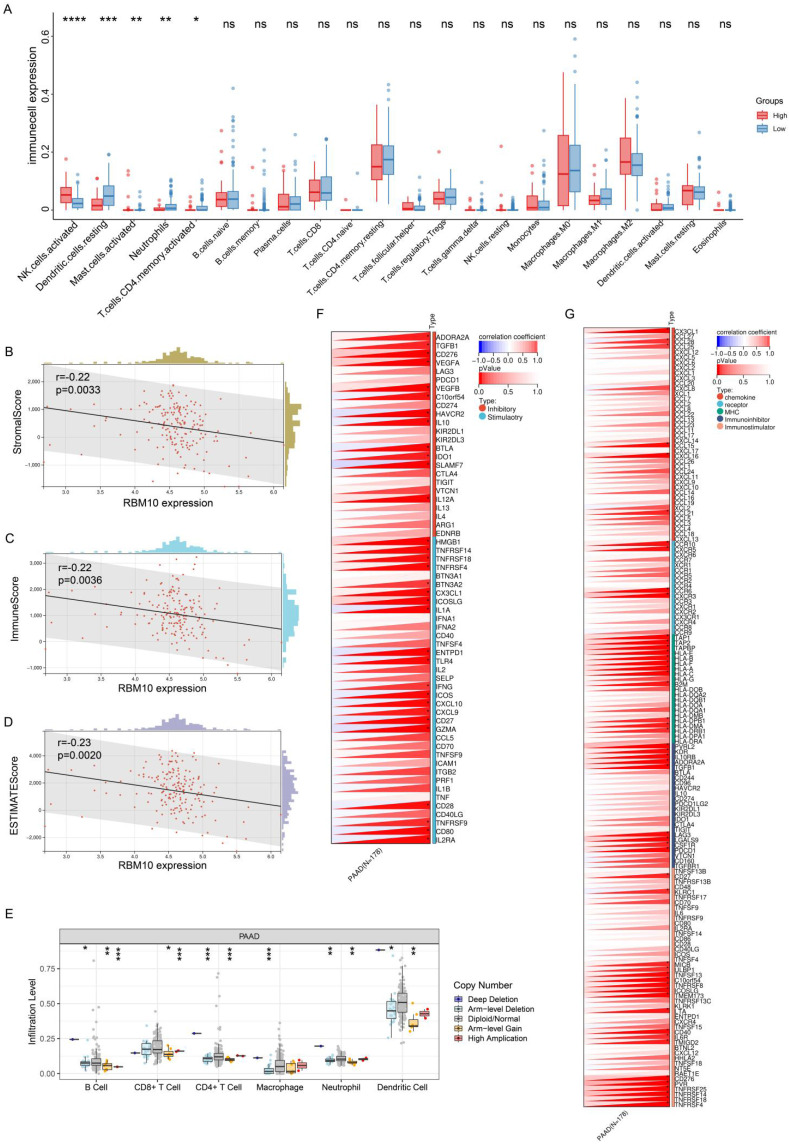
** Immunological characterization of RBM10 in PAAD. A.** Correlation between RBM10 expression and infiltration of different immune cells. **B.** Correlation between RBM10 expression and Stromal Score.** C.** Correlation between RBM10 expression and Immune Score.** D.** Correlation between RBM10 expression and ESTIMATE Score.** E.** Correlation between RBM10 gene alterations (e.g., copy number variations) and immune cell infiltration, analysed using TIMER. **F.** Correlation between RBM10 expression and immune checkpoint genes. **G.** Correlation between RBM10 expression and immunomodulatory genes.

To understand the relationship between RBM10 and immune infiltration in pancreatic adenocarcinoma (PAAD), we analysed PAAD data from the TCGA database. Using the CIBERSORTx online tool (https://cibersortx.stanford.edu/), we assessed immune cell infiltration and visualized the results using R software. This analysis revealed that RBM10 expression was associated with the infiltration of NK cells, dendritic cells (DCs), mast cells, neutrophils, and CD4^+^ T cells (Figure 5A). Among these cells, the difference in NK cell infiltration was most pronounced, with significantly higher infiltration in the RBM10 high-expression group compared to the RBM10 low‑expression group (Figure 5A).

Furthermore, using the immune-infiltration analysis module of Sangerbox (http://sangerbox.com/), we obtained "StromalScore," "ImmuneScore," and "ESTIMATEScore." RBM10 expression was found to be significantly negatively correlated with all three scores (Figure 5B-D).

Next, we applied TIMER (https://cistrome.shinyapps.io/timer/), an online tool, to analyse the relationship between RBM10 gene copy number and immune cell infiltration in PAAD. The results demonstrated a correlation between RBM10 copy number alterations and the infiltration levels of multiple immune cell types (Figure 5E).

Finally, we examined the relationship between RBM10 expression and immune checkpoint genes as well as immunoregulatory genes, identifying significant correlations with multiple genes in both categories (Figure 5F, G).

### Low RBM10 expression affected PD-1 expression on NK cells

**Figure 6:**
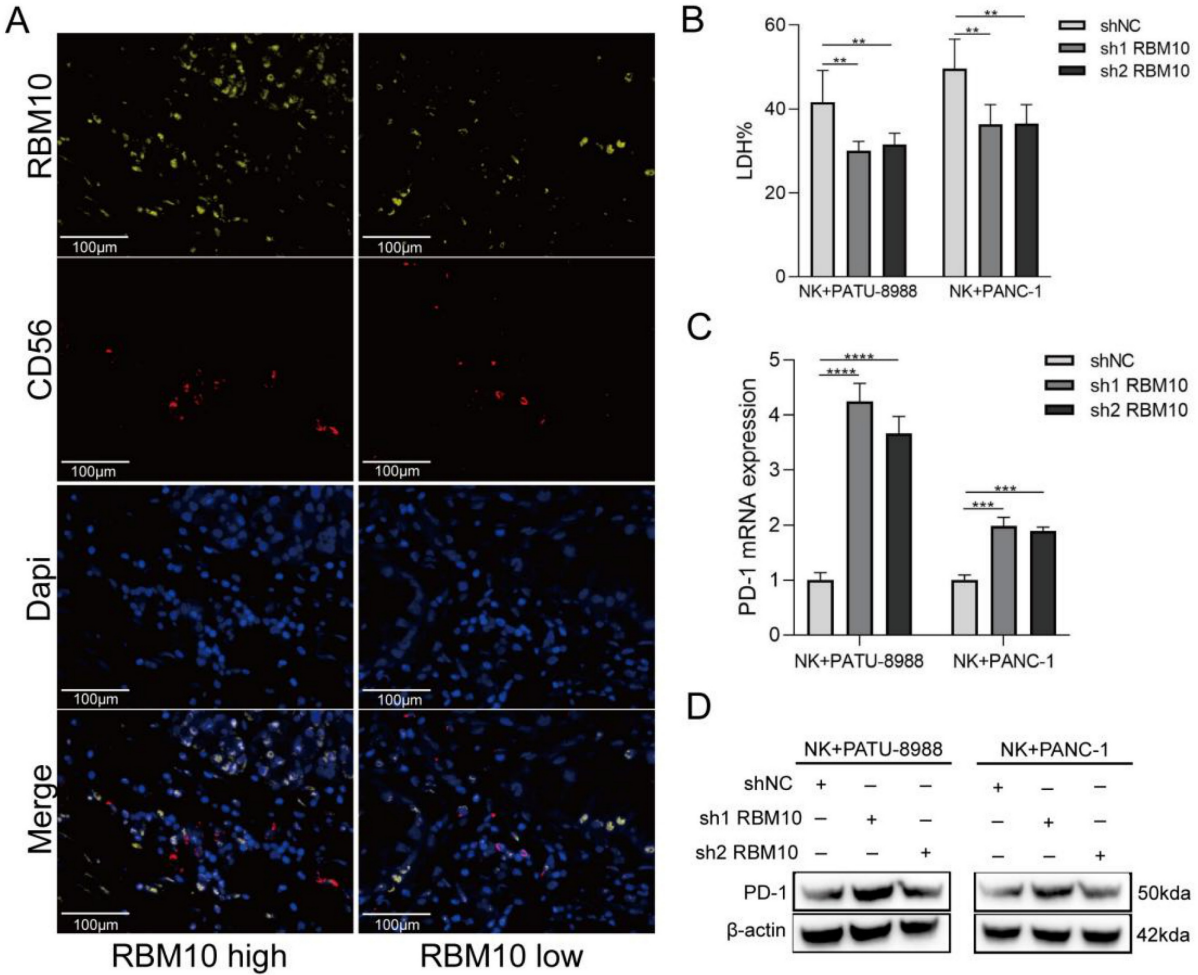
** Low RBM10 expression affects PD-1 expression on NK cell. A.** mIHC staining results for RBM10 and CD56 expression in the tissues from PAAD patients;** B.** Cytotoxicity of NK92MI cells to PAAD cells, detected by the LDH assay; **C-D.** RT-qPCR (C) and WB (D) analyses of PD-1 expression in NK92MI cells after co-culture.

Immunoinfiltration analysis revealed a significant association between high RBM10 expression and increased NK cell infiltration in PAAD samples (Figure 5A). The data revealed a correlation between CD56 and RBM10 expression: CD56 expression was decreased in the tissues from PAAD patients with low RBM10 expression compared to those with high RBM10 expression (Figure 6A).

Next, we co-cultured RBM10-knockdown PAAD cells with NK92MI (NK cell line) cells. The killing effect of NK92MI cells on the PAAD cells was significantly attenuated (~20%, P < 0.05) after RBM10 knockdown compared to the control group (Figure 6B). Additionally, we measured the PD‑1 expression level on NK92MI cells after co-culture, using RT-qPCR and Western blot, showing that PD-1 expression was upregulated in NK92MI cells after co-culture (Figure 6C, D).

### Low RBM10 expression promoted NK cell depletion through the JAK-STAT pathway

**Figure 7:**
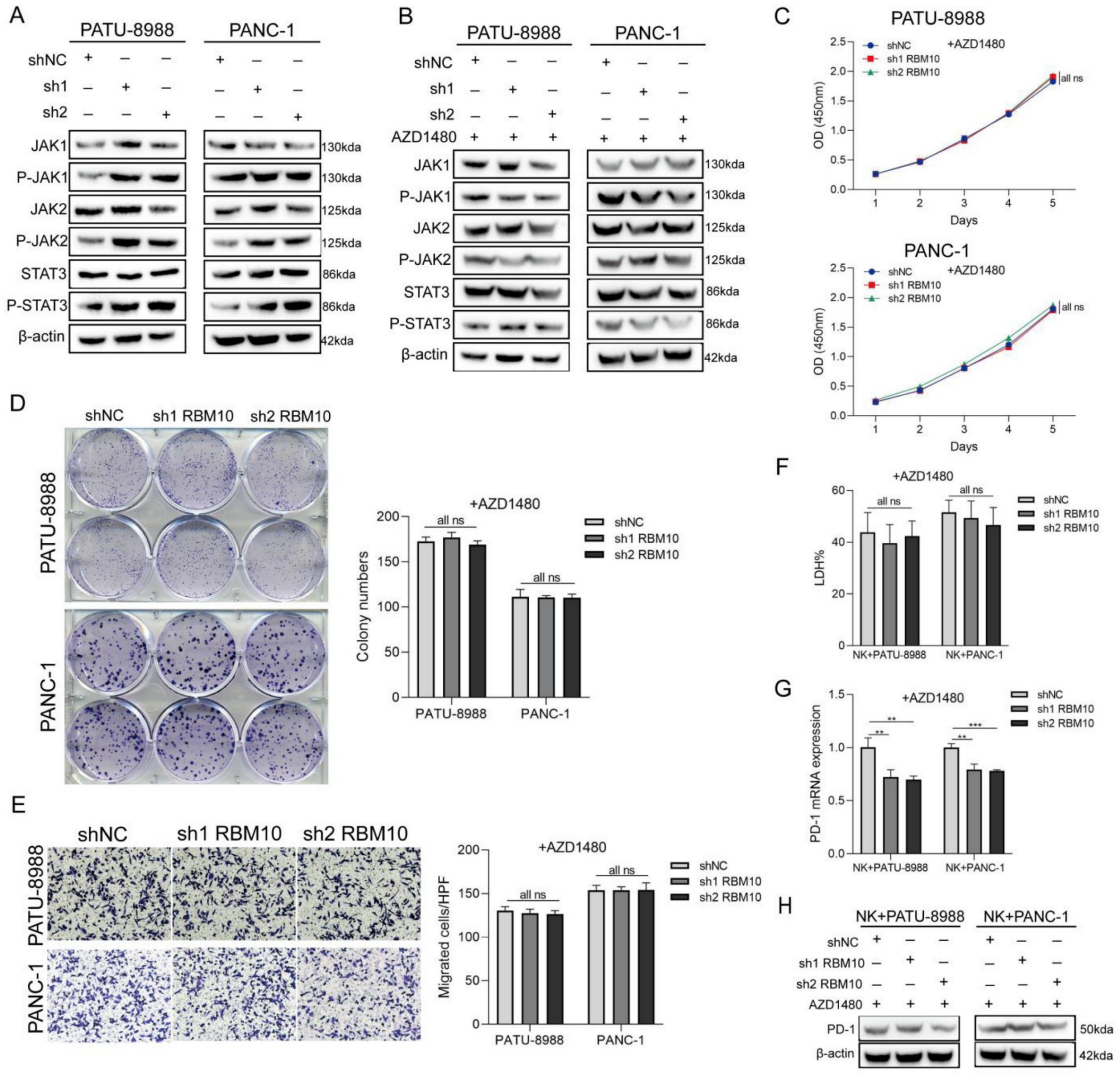
** Low RBM10 expression promoted NK cell depletion through the JAK-STAT pathway. A.** Western blot (WB) detection of JAK-STAT pathway-related protein expression in PATU-8988 and PANC-1 cells after RBM10 knockdown. **B.** WB detection of JAK-STAT pathway-related protein expression in PATU-8988 and PANC-1 cells after RBM10 knockdown and treatment with the JAK pathway inhibitor AZD1480. **C.** CCK-8 assay of PATU-8988 and PANC-1 cells after RBM10 knockdown and treatment with AZD1480. **D.** Colony formation assays of PATU-8988 and PANC-1 cells after RBM10 knockdown and treatment with AZD1480. **E.** Transwell assay of PATU-8988 and PANC-1 cells after RBM10 knockdown and treatment with AZD1480. **F.** The cytotoxicity of NK92MI cells against PAAD cells, detected by the LDH method, in co-culture after treatment with AZD1480. **G-H.** RT-qPCR (G) and WB (H) detection of PD-1 expression in NK92MI cells after AZD1480 treatment in the co-culture system.

Given the significant alterations in the JAK-STAT pathway observed in our GSEA analysis, and considering that activation of JAK1 and JAK2 inhibits tumour cell susceptibility to NK cells by upregulating PD-L1 expression 39, we examined changes in the JAK-STAT signalling pathway in RBM10 knockdown cell lines. Western blot analysis showed that the expression of P-JAK1, P-JAK2, and P-STAT3 was upregulated in both PATU-8988 and PANC-1 cells following RBM10 knockdown (Figure 7A).

To determine whether regulation of the JAK-STAT pathway is essential, we added the JAK pathway inhibitor AZD1480 to the culture medium of RBM10-knockdown pancreatic cancer cells *in vitro*. The results demonstrated that the significant increase in cellular proliferation and migration observed with RBM10 knockdown was attenuated following AZD1480 treatment (Figure 7B-E). Similarly, AZD1480 restored the killing effect of NK92MI cells on RBM10-knockdown PAAD cells in co-culture (Figure 7F). This restoration was consistent with a reduction in PD-1 expression on NK cells, as shown by RT-qPCR and WB analysis (Figures 7G, H).

## Discussion

Pancreatic cancer has a poor prognosis, with a 5-year survival rate of less than 10% 1, 2. This is primarily due to its insidious onset and the lack of reliable early diagnostic approaches with high sensitivity and specificity. Thus, identifying novel biomarkers, such as RBM proteins, is essential for improving early detection and patient outcomes.

RBM proteins, which are involved in various RNA metabolism processes, exhibit aberrant expression and dysfunction closely linked to cancer development 7. RBM10, an important member of the RBM family, initially gained attention due to its association with TARP syndrome, a rare X-linked genetic disorder caused by mutations in the RBM10 gene 40. RBM10 plays diverse roles in cancer development; for example, targeting RBM10 promotes the progression of endometrial cancer, whereas it suppresses the development of cholangiocarcinoma 41. These findings highlight the context-dependent roles of RBM10 in tumour biology.

Our current study demonstrated reduced RBM10 expression in human pancreatic cancer tissues. Notably, high RBM10 expression was associated with improved prognosis, suggesting a tumour‑suppressive role for RBM10. This observation is supported by our study *in vitro*, showing that RBM10 knockdown increases pancreatic cancer cell viability, colony formation, and migration. These findings underscore RBM10's critical role in regulating pancreatic cancer cell proliferation and migration.

To investigate RBM10's function in disease further, we studied RBM10 knockdown mice. These mice exhibited a localized decrease in alveoli (air sacs) and an increase in glandular ductal epithelium (tissue lining ducts). Additionally, we observed mild fibrosis (scarring) and inflammatory cell infiltration, which worsened with age. These findings suggest that RBM10 is crucial for normal pancreatic development. Our data align with reports showing RBM10 mutations cause TARP syndrome, which affects heart, brain, and limb development 42. This dysregulation is likely mediated by RBM10's interaction with RNA near splice sites, potentially leading to either loss or gain of function 43.

Despite these observations, when we followed up on RBM10-knockdown mice for up to 9 months (approximately equivalent to 30 years in humans), no cancerous or precancerous lesions were detected in the pancreas at the histopathological level. This may be due to the relatively short lifespan of mice compared to humans (i.e., months vs years). Since malignancy is often associated with age, it is understandable that tumour development may not have occurred within the study period. To further investigate RBM10's potential role in pancreatic cancer development, we propose studying this protein in larger animal models, such as genetically modified rabbits or sheep, if feasible 44.

Bioinformatics revealed that RBM10 expression is closely correlated with immune cell infiltration, particularly NK cell infiltration 31. Consistent with these findings, our study identified significantly supressed RBM10 expression in pancreatic cancer tissues, accompanied by decreased NK cell infiltration. Considering that NK cells play a crucial role in host immunosurveillance against malignancy and are integral to tumour immunotherapy 45, these observations suggest that host immunity against malignancy may be compromised due to suppressed RBM10 expression in the pancreatic tissues of a susceptible cohort. However, it remains unclear whether reduced RBM10 expression leads to or results from inhibited NK cell infiltration in pancreatic cancer. Thus, further studies clarifying the relationship between RBM10 and NK cell infiltration, particularly its impact on cytotoxicity *in vivo* and *in vitro*, could provide valuable insights for developing novel targeted therapies for pancreatic cancer 46.

Subsequently, to confirm the effector function of NK cells against pancreatic cancer, particularly in the context of RBM10 expression, we co-cultured NK cells with RBM10-knockdown pancreatic cancer cells. We observed attenuated killing of RBM10-knockdown pancreatic cancer cells by NK cells, accompanied by upregulated PD-1 expression. These findings suggest that RBM10 contributes to host innate immunity (NK cells) against pancreatic cancer development through modulation of the PD-1 pathway. Our data further imply a close interaction between RBM10 expression and the PD-1 pathway in cancer development. However, a report shows that high RBM10 expression and PD-L1 positivity in patients with lung adenocarcinoma are associated with poor outcomes 47, suggesting differential role of RBM10 in the tumorigenesis of different tumours. It should be noted that the microenvironments of the lung and pancreas are almost completely different and may initiate distinct host immune responses, which will be clarified in the future.

Given that activation of the JAK1 and JAK2 pathways has been demonstrated to inhibit tumour cell susceptibility to NK cells by upregulating PD-L1 expression 39, we further investigated the activation status of the JAK-STAT pathway in RBM10-knockdown pancreatic cancer cells. Our results confirmed that the expression of phosphorylated JAK1 (P-JAK1), phosphorylated JAK2 (P-JAK2), and phosphorylated STAT3 (P-STAT3) was upregulated in pancreatic cancer cells following RBM10 knockdown. Notably, the altered proliferation and migration observed in RBM10‑knockdown cells were reversed after treatment with the JAK pathway inhibitor (AZD1480). Additionally, the killing effect of NK cells on RBM10-knockdown pancreatic cancer cells was restored in response to treatment with the JAK pathway inhibitor. This was accompanied by a decrease in PD-1 expression, as confirmed by both RT-qPCR (for mRNA levels) and Western blotting (for protein levels). Our data confirm the functional interaction among NK cells, RBM10, and the (P-JAK1, P-JAK2, P-STAT3) signalling pathways.

Our findings are consistent with reports showing that JAK-STAT-mediated chronic inflammation in pancreatic cancer impairs cytotoxic T-lymphocyte activation, thereby reducing the effectiveness of anti-PD-1 immunotherapy 48. Significant changes in the JAK-STAT pathway were also observed in our GSEA analysis. These results reinforce the link between the JAK-STAT pathway, NK cell activity, and pancreatic cancer progression, as confirmed in our *in vitro* studies.

Xiao *et al.* demonstrated that RBM10 regulates hTERT splicing 22; however, its impact on pancreatic cancer patient survival was not explored. Our study specifically focuses on RBM family members associated with pancreatic cancer patient survival, while we acknowledge that subsequent experiments also involved hTERT splicing. The most important aspect of our study is its exploration of immune-related host responses using bioinformatics approaches, ultimately identifying NK cells as key targets for further investigation. Furthermore, we validated the effect of RBM10 deletion on NK cells, substantiating our findings through complementary experiments.

Additionally, Wu *et al.* investigated NPTX1 in relation to RBM10's role in enhancing drug sensitivity but did not conduct a mechanistic analysis 23. However, the cellular phenotypic changes associated with RBM10 have not been verified, indicating a potential interaction between NPTX1 and RBM10. Our study explores the relationship between RBM10 and pancreatic cancer immunity, showing that RBM10 loss may influence PD-1 expression in NK cells through the JAK-STAT pathway, potentially contributing to tumour immune escape. Understanding the mechanistic interactions between RBM10, the JAK-STAT pathway, and NK cell function provides a promising avenue for developing targeted therapies for pancreatic cancer.

However, direct evidence for RBM10's role in regulating host immunity, particularly its linkage with NK cell function in malignancy *in vivo*, remains to be fully elucidated. This will be further investigated in our future studies.

There are limitations to the current study. First, we did not investigate in depth the mechanism by which RBM10 affects PD-1 expression in NK cells, which may become the focus of our future research. Second, no neoplastic lesions were detected in RBM10-manipulated 9 months old mice. This could be attributed to their relatively short lifespan or the absence of key environmental stimuli (e.g., activation of the JAK-STAT pathway or PD-1 expression) required for the development of pancreatic cancer.

## Conclusion

In conclusion, our findings suggest that RBM10 deletion alters the JAK-STAT pathway, leading to increased PD-1 expression in NK cells. This, in turn, contributes to NK cell depletion and promotes tumour progression. Understanding the mechanistic interplay between RBM10, the JAK-STAT pathway, and NK cell function offers a promising avenue for the development of targeted therapies to manage pancreatic cancer.

## Supplementary Material

Supplementary figure and tables.

## References

[1] Mizrahi JD, Surana R, Valle JW, Shroff RT (2020). Pancreatic cancer. Lancet.

[2] Kleeff J, Korc M, Apte M, La Vecchia C, Johnson CD, Biankin AV (2016). Pancreatic cancer. Nat Rev Dis Primers.

[3] Del Chiaro M, Sugawara T, Karam SD, Messersmith WA (2023). Advances in the management of pancreatic cancer. Bmj.

[4] Hu ZI, O'Reilly EM (2024). Therapeutic developments in pancreatic cancer. Nat Rev Gastroenterol Hepatol.

[5] Brozos-Vázquez E, Toledano-Fonseca M, Costa-Fraga N, García-Ortiz MV, Díaz-Lagares Á, Rodríguez-Ariza A (2024). Pancreatic cancer biomarkers: A pathway to advance in personalized treatment selection. Cancer Treat Rev.

[6] Wood LD, Canto MI, Jaffee EM, Simeone DM (2022). Pancreatic Cancer: Pathogenesis, Screening, Diagnosis, and Treatment. Gastroenterology.

[7] Li Z, Guo Q, Zhang J, Fu Z, Wang Y, Wang T (2021). The RNA-Binding Motif Protein Family in Cancer: Friend or Foe?. Front Oncol.

[8] Su Z, Wang K, Li R, Yin J, Hao Y, Lv X (2016). Overexpression of RBM5 induces autophagy in human lung adenocarcinoma cells. World J Surg Oncol.

[9] Guan B, Li G, Wan B, Guo X, Huang D, Ma J (2021). RNA-binding protein RBM38 inhibits colorectal cancer progression by partly and competitively binding to PTEN 3'UTR with miR-92a-3p. Environ Toxicol.

[10] Feng H, Liu J, Qiu Y, Liu Y, Saiyin H, Liang X (2020). RNA-binding motif protein 43 (RBM43) suppresses hepatocellular carcinoma progression through modulation of cyclin B1 expression. Oncogene.

[11] Han H, Lin T, Wang Z, Song J, Fang Z, Zhang J (2023). RNA-binding motif 4 promotes angiogenesis in HCC by selectively activating VEGF-A expression. Pharmacol Res.

[12] Xi PW, Zhang X, Zhu L, Dai XY, Cheng L, Hu Y (2020). Oncogenic action of the exosome cofactor RBM7 by stabilization of CDK1 mRNA in breast cancer. NPJ Breast Cancer.

[13] Inoue A, Yamamoto N, Kimura M, Nishio K, Yamane H, Nakajima K (2014). RBM10 regulates alternative splicing. FEBS Lett.

[14] Jin X, Di X, Wang R, Ma H, Tian C, Zhao M (2019). RBM10 inhibits cell proliferation of lung adenocarcinoma via RAP1/AKT/CREB signalling pathway. J Cell Mol Med.

[15] Nanjo S, Wu W, Karachaliou N, Blakely CM, Suzuki J, Chou YT (2022). Deficiency of the splicing factor RBM10 limits EGFR inhibitor response in EGFR-mutant lung cancer. J Clin Invest.

[16] Bao Y, Zhang S, Zhang X, Pan Y, Yan Y, Wang N (2023). RBM10 Loss Promotes EGFR-Driven Lung Cancer and Confers Sensitivity to Spliceosome Inhibition. Cancer Res.

[17] Zhao J, Sun Y, Huang Y, Song F, Huang Z, Bao Y (2017). Functional analysis reveals that RBM10 mutations contribute to lung adenocarcinoma pathogenesis by deregulating splicing. Sci Rep.

[18] Guo L, Wang Y, Yang W, Wang C, Guo T, Yang J (2023). Molecular Profiling Provides Clinical Insights into Targeted and Immunotherapies as Well as Colorectal Cancer Prognosis. Gastroenterology.

[19] Zhao Z, Li J, Shen F (2020). Protective effect of the RNA-binding protein RBM10 in hepatocellular carcinoma. Eur Rev Med Pharmacol Sci.

[20] Li Y, Wei D, Chen Z, Chen Y, Deng Y, Li M (2024). RBM10 regulates the tumorigenic potential of human cancer cells by modulating PPM1B and YBX1 activities. Exp Cell Res.

[21] Cao Y, Di X, Zhang Q, Li R, Wang K (2021). RBM10 Regulates Tumor Apoptosis, Proliferation, and Metastasis. Front Oncol.

[22] Xiao W, Chen X, Li X, Deng K, Liu H, Ma J (2021). RBM10 regulates human TERT gene splicing and inhibits pancreatic cancer progression. Am J Cancer Res.

[23] Wu J, Liu G, An K, Shi L (2022). NPTX1 inhibits pancreatic cancer cell proliferation and migration and enhances chemotherapy sensitivity by targeting RBM10. Oncol Lett.

[24] Mainous AG 3rd, Struelens MJ, Bao S (2024). The importance of patients in conflict of interest declarations. Front Med (Lausanne).

[25] Taylor SC, Posch A (2014). The design of a quantitative western blot experiment. Biomed Res Int.

[26] Liu Y, Liu P, Duan S, Lin J, Qi W, Yu Z (2025). CTCF enhances pancreatic cancer progression via FLG-AS1-dependent epigenetic regulation and macrophage polarization. Cell Death Differ.

[27] Wu L, Liu F, Yin L, Wang F, Shi H, Zhao Q (2022). The establishment of polypeptide PSMA-targeted chimeric antigen receptor-engineered natural killer cells for castration-resistant prostate cancer and the induction of ferroptosis-related cell death. Cancer Commun (Lond).

[28] Liu P, Gao X, Yu Z, Liu Y, Liu Y, Lin J (2024). H19 promotes polarization and alternative splicing in tumor-associated macrophages, facilitating pancreatic cancer progression. Cancer Lett.

[29] Wu B, Hu K, Li S, Zhu J, Gu L, Shen H (2012). Dihydroartiminisin inhibits the growth and metastasis of epithelial ovarian cancer. Oncol Rep.

[30] Ye H, Yu W, Li Y, Bao X, Ni Y, Chen X (2023). AIM2 fosters lung adenocarcinoma immune escape by modulating PD-L1 expression in tumor-associated macrophages via JAK/STAT3. Hum Vaccin Immunother.

[31] Newman AM, Steen CB, Liu CL, Gentles AJ, Chaudhuri AA, Scherer F (2019). Determining cell type abundance and expression from bulk tissues with digital cytometry. Nat Biotechnol.

[32] Shen W, Song Z, Zhong X, Huang M, Shen D, Gao P (2022). Sangerbox: A comprehensive, interaction-friendly clinical bioinformatics analysis platform. Imeta.

[33] Li T, Fan J, Wang B, Traugh N, Chen Q, Liu JS (2017). TIMER: A Web Server for Comprehensive Analysis of Tumor-Infiltrating Immune Cells. Cancer Res.

[34] Zhang X, Yuan L, Tan Z, Wu H, Chen F, Huang J (2024). CD64 plays a key role in diabetic wound healing. Front Immunol.

[35] Yuan L, Tan Z, Huang J, Chen F, Hambly BD, Bao S (2024). Exploring the clinical significance of IL-38 correlation with PD-1, CTLA-4, and FOXP3 in colorectal cancer draining lymph nodes. Front Immunol.

[36] Wu H, Yang J, Yuan L, Tan Z, Zhang X, Hambly BD (2024). IL-38 promotes the development of prostate cancer. Front Immunol.

[37] Zhang W, Hubbard A, Jones T, Racolta A, Bhaumik S, Cummins N (2017). Fully automated 5-plex fluorescent immunohistochemistry with tyramide signal amplification and same species antibodies. Lab Invest.

[38] Chen F, Zhang F, Tan Z, Hambly BD, Bao S, Tao K (2020). Interleukin-38 in colorectal cancer: a potential role in precision medicine. Cancer Immunol Immunother.

[39] Bellucci R, Martin A, Bommarito D, Wang K, Hansen SH, Freeman GJ (2015). Interferon-γ-induced activation of JAK1 and JAK2 suppresses tumor cell susceptibility to NK cells through upregulation of PD-L1 expression. Oncoimmunology.

[40] Daicheng H, Shiwen X, Jingxuan Z, Junbo H, Bo W (2022). A Frameshift RBM10 Variant Associated with TARP Syndrome. Front Genet.

[41] Chang J, Zhang Y, Zhou T, Qiao Q, Shan J, Chen Y (2024). RBM10 C761Y mutation induced oncogenic ASPM isoforms and regulated β-catenin signaling in cholangiocarcinoma. J Exp Clin Cancer Res.

[42] Niceta M, Barresi S, Pantaleoni F, Capolino R, Dentici ML, Ciolfi A (2019). TARP syndrome: Long-term survival, anatomic patterns of congenital heart defects, differential diagnosis and pathogenetic considerations. Eur J Med Genet.

[43] Wang Y, Gogol-Döring A, Hu H, Fröhler S, Ma Y, Jens M (2013). Integrative analysis revealed the molecular mechanism underlying RBM10-mediated splicing regulation. EMBO Mol Med.

[44] Hou Y, Zhang X, Sun X, Qin Q, Chen D, Jia M (2022). Genetically modified rabbit models for cardiovascular medicine. Eur J Pharmacol.

[45] Myers JA, Miller JS (2021). Exploring the NK cell platform for cancer immunotherapy. Nat Rev Clin Oncol.

[46] Jiang P, Jing S, Sheng G, Jia F (2024). The basic biology of NK cells and its application in tumor immunotherapy. Front Immunol.

[47] Isaka T, Miyagi Y, Yokose T, Saito H, Kasajima R, Watabe K (2023). Impact of RBM10 and PD-L1 expression on the prognosis of pathologic N1-N2 epidermal growth factor receptor mutant lung adenocarcinoma. Transl Lung Cancer Res.

[48] Lu C, Talukder A, Savage NM, Singh N, Liu K (2017). JAK-STAT-mediated chronic inflammation impairs cytotoxic T lymphocyte activation to decrease anti-PD-1 immunotherapy efficacy in pancreatic cancer. Oncoimmunology.

