# Supplementary material for: RBM10 inhibits pancreatic cancer development by suppressing immune escape through PD-1 expression
Source: J Cancer. 2025 Jul 4;16(10):3080–93. doi: 10.7150/jca.111459 (PMC12305579; doi:10.7150/jca.111459)
Supplement: Supplementary file 1 — Supplementary figure and tables. [file jcav16p3080s1.pdf]

Supplementary material

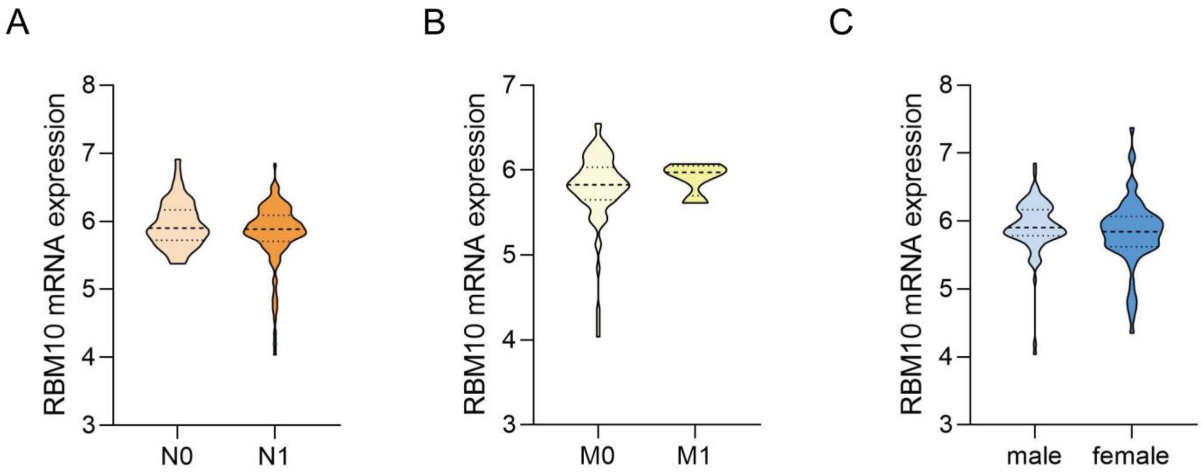

Figure S1.

A-C. Clinicopathologic features of PAAD associated with RBM10 expression in the TCGA database: N stage (A), M stage (B), Gender (C).

Table S1 List of primer sequences

gene	Forward Primer Sequence 5’−3’	Forward Primer Sequence 5’−3’
RBM10	ATGGAGTATGAAAGACGTGGTGG	TCCCGGTAGTCGTGGTCTC
RBM10-mouse	GTCACTTGCAGGACGCTACA	CACACTGTGAGGCACACTTAT
PD-1	CCAGGATGGTTCTTAGACTCCC	TTTAGCACGAAGCTCTCCGAT
GAPDH	ACAACTTTGGTATCGTGGAAGG	GCCATCACGCCACAGTTTC
GAPDH-mouse	AGGTCGGTGTGAACGGATTTG	GGGGTCGTTGATGGCAACA

Table S2 List of Antibody Information

Antibody	Production company	Catalogue number	Dilution ratio
RBM10	Abcam	ab224149	1:10000
PD-1	proteintech	66220-1-Ig	1:5000
CD56	Servicebio	GB12041-50	1:600
JAK1	CST	3344T	1:1000
P-JAK1	CST	74129T	1:1000
JAK2	CST	3230T	1:1000
P-JAK2	CST	4406T	1:1000
STAT3	CST	9139T	1:1000
P-STAT3	CST	9145T	1:2000
β-actin	ABclonal	AC038	1:10000

Table S3 List of Plasmid sequences

ID	Plasmid sequences
sh1 RBM10	5’-GACATGGACTACCGTTCATAT-3’
sh2 RBM10	5’-CTTCGCCTTCGTCGAGTTTAG-3’
